# Characterization of conformation-specific, human-derived monoclonal antibodies against TTR aggregates with potential for diagnostic and therapeutic use

**DOI:** 10.1186/1750-1172-10-S1-P39

**Published:** 2015-11-02

**Authors:** Aubin Michalon, Benoit Combaluzier, Evita Varela, Andreas Hagenbuch, Ole Suhr, Maria Saraiva, Jan Grimm

**Affiliations:** 1 Neurimmune, 8952, Schlieren, Switzerland; 2 Umeå University, 90187, Umeå, Sweden; 3 IBMC, 4150, Porto, Portugal

## 

Misfolding and aggregation of transthyretin (TTR) is the basic pathophysiological mechanism of hereditary and wild type TTR amyloid (ATTR) amyloidosis. Polyneuropathy and/or cardiomyopathy with heart failure dominates the clinical presentation of the disease. Conformational changes of the TTR protein structure produce toxic intermediates that introduce cell death and ultimately loss of organ function. Reliable and early diagnosis of ATTR amyloidosis are, however, difficult to obtain based on the patients’ clinical presentation, since different types of neuropathies or cardiomyopathies often exhibit similar clinical findings, especially early after onset of disease.

Since misfolded TTR aggregates constitutes an early phase of amyloid formation, availability of a diagnostic tests able to detect misfolded TTR aggregates either biochemically or by imaging techniques would facilitate a correct and early diagnosis of ATTR amyloidosis.

By comprehensive genetic sequence analyses of the human immune repertoire for TTR-specific memory B-cells, we cloned and recombinantly expressed human-derived monoclonal antibodies, which specifically bind with high affinity and selectivity to misfolded TTR but not physiological TTR tetramers. These antibodies specifically detect ATTR deposits in ATTR amyloidosis patients’ biopsies and TTR aggregates in corresponding tissues from TTR transgenic mouse-models. The selectivity of the human antibodies for misfolded TTR is driven by their targeting of cryptic or conformational epitopes which are exposed in misfolded and/or aggregated wild type as well as mutant TTR.

These conformation-specific, human-derived monoclonal antibodies are promising candidates for diagnostic use, and for the development of disease-modifying therapies for TTR amyloidosis by targeting and facilitating the removal of disease-causing TTR aggregates.

